# “*Dosis sola facit venenum*”—Evidence for causality in the association between ketamine and cholestatic liver injury

**DOI:** 10.1007/s12072-024-10646-w

**Published:** 2024-03-13

**Authors:** Pedro David Wendel-Garcia, Rea Andermatt, Christian Bode, Sascha David, Klaus Stahl

**Affiliations:** 1 Institute of Intensive Care Medicine, University Hospital Zurich, Zurich, Switzerland; 2 Department of Anesthesiology and Intensive Care Medicine, University Hospital Bonn, Bonn, Germany; 3 Department of Nephrology, Hannover Medical School, Hannover, Germany; 4 Department of Gastroenterology, Hepatology, Infectious Diseases and Endocrinology, Hannover Medical School, Carl-Neuberg Straße 1, 30163 Hannover, Germany

**Keywords:** ARDS, SSC-CIP, Sclerosing cholangitis, Liver injury

To the Editor,

With great interest, we read the article by Leonhardt et al. [1] describing diverse risk factors for developing secondary sclerosing cholangitis (SSC) following severe Covid-19.

Although biliary injury in critically ill patients is most likely multifactorial, the use of intravenous ketamine has been suggested to potentially contribute to its pathophysiology [2, 3]. In the present analysis use of ketamine was significantly associated with later onset of SSC. The authors, however, question the causality of ketamine in relation to consequent cholestatic injury as the underlying biological mechanisms for this remain unclear. They therefore speculate that this correlation might rather be due to further unknown confounding factors.

The quest to uncover the causality between ketamine and biliary injury is not much different from the original undertaking of Bradford Hill and colleagues to uncover the causal association between cigarette smoking and lung cancer brought forward by Hill in 1965 [4]. Given those principles, not (yet) knowing the exact biological mechanism of ketamine causing SSC, does not invalidate the causal relationship between ketamine and cholestatic liver injury itself. In the words of Hill himself, “what is biologically plausible depends upon the biological knowledge of the day”.

Key to postulate a causal effect resides instead in unravelling a few, but critical links in the causal constellation between exposure and outcome. Most importantly there are no clear-cut rules or “sine qua non” requirements for causality; contrarily, causation is fundamentally interpretative. However, in this case, we have evidence for the strength, consistency, specificity, temporality, biological gradient, epidemiological plausibility, coherence and reversibility of the association between ketamine and cholestatic liver injury and are merely missing a biological pathway (Fig. 1).

**Fig. 1:**
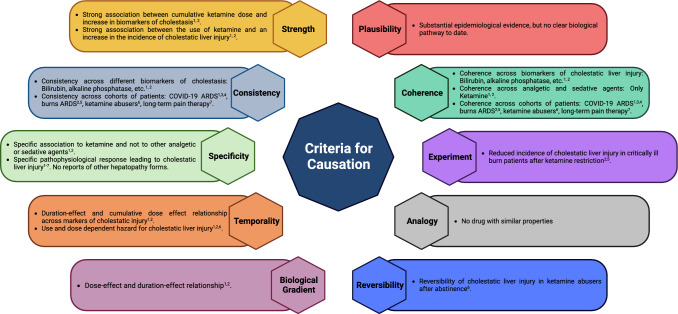
Bradford Hill criteria for causation, and the available evidence linking ketamine as an exposure to cholestatic liver injury as an outcome. These criteria have to be understood as a framework to deduce causality from association. However, the postulation of causality remains a fundamentally interpretative task without clear-cut rules

Focusing on a single aspect, a major determinant of causality not considered by the authors, is the dose–effect relationship of a drug, or as Paracelsus described it more than 450 years ago *“Dosis sola facit venenum”.* In fact, our group has demonstrated in a retrospective cohort of COVID-19 ARDS patients, that intravenous use of ketamine was both dose- and time-dependently associated with the onset of significant cholestatic liver injury, which was not observed for propofol or opioids [3]. Similarly, others showed a dose-dependent association between ketamine and the overall incidence of cholestatic injury in critically ill burn patients, not seen for midazolam or sufentanil [5]. Importantly, the legal restriction of ketamine use in France leads to a strong reduction in the incidence of cholestatic liver injury in critically ill burn patients [2, 5].

No one would negate the causative effect of cigarette smoking on the inception of lung cancer nowadays, even though no randomized evidence or clear biological pathway existed at the time it was first described.

Although we cannot completely rule out other confounding factor(s) that may also cause SSC in critically ill patients, fairly strong evidence does now exist for the long-term, high-dose use of ketamine that should prompt us to reassess its adequacy for critically ill patients. After all, if we are wrong in deducing causation from associative data in this case, no great harm will be done, as opposed to much potential good.

## References

[1] Leonhardt S, Jürgensen C, Frohme J (2023). Hepatobiliary long-term consequences of COVID-19: dramatically increased rate of secondary sclerosing cholangitis in critically ill COVID-19 patients. Hepatol Int.

[2] de Tymowski C, Dépret F, Dudoignon E (2021). Ketamine-induced cholangiopathy in ARDS patients. Intensive Care Med.

[3] Wendel-Garcia PD, Erlebach R, Hofmaenner DA (2022). Long-term ketamine infusion-induced cholestatic liver injury in COVID-19-associated acute respiratory distress syndrome. Crit Care.

[4] Hill AB (2015). The environment and disease: association or causation?. J R Soc Med.

[5] De Tymowski C, Dépret F, Dudoignon E, et al. Ketamine restriction correlates with reduced cholestatic liver injury and improved outcomes in critically ill burn patients. JHEP Rep. 2023;6(2):100950. 10.1016/j.jhepr.2023.100950 PMC10832380 38304235

